# Deep learning and dual-radiomics model incorporating brachytherapy applicator type to predict radiation-induced acute rectal injury in cervical cancer patients

**DOI:** 10.1016/j.phro.2026.100908

**Published:** 2026-01-20

**Authors:** Boda Ning, Zhengxian Li, Deyang Yu, Chenyu Li, Qi Liu, Yanling Bai

**Affiliations:** a Department of Radiation Physics, Harbin Medical University Cancer Hospital, Harbin 150081, China; b Radiotherapy Center, Jilin Guowen Hospital, Changchun 130000, China; c Department of Radiology, Xiangya Hospital of Central South University, Changsha 410000, China

**Keywords:** Radiation-induced acute rectal injury, Cervical cancer, Radiomics, Deep learning

## Abstract

**Background and purpose:**

Radiation-induced acute rectal injury (RARI) is a common early toxicity after radiotherapy for cervical cancer (CC) and remains difficult to predict before treatment, which can adversely affect life quality of patients. We aimed to develop a combined dual-radiomics and deep learning (DL) model to improve the prediction of RARI in CC patients treated with radiotherapy.

**Materials and methods:**

This retrospective study included 200 CC patients from one hospital, randomly divided into training (n = 160), internal validation (n = 40) cohorts and external validation (n = 40) from another hospital. Patients were classified as RARI (CTCAE v5.0 grade ≥ 2) or Non-RARI (grade < 2). Radiomic and dosiomic features were extracted from CT images and dose distributions, and DL features were learned using 3D CNNs. The performance of radiomics, dosiomics, DL and hybrid features models for RARI prediction was compared using the receiver operating characteristic (ROC) curve with measurement of the area under the curve (AUC).

**Results:**

For radiomics combining dosiomics, XGBoost achieved the best performance with AUCs of 0.786 and 0.755 in internal and external validation cohorts, respectively. For DL, Resnet_with_CBAM achieved the best performance in the input of combining CT and dose distribution with AUCs of 0.786 and 0.773 in internal and external validation cohorts, respectively. Nomogram integrating radiomics, dosiomics, DL features, and clinical factor improved the AUC to 0.810, 0.803 in internal and external validation cohorts, respectively.

**Conclusion:**

The nomogram integrating radiomics, dosiomics, DL, and clinical factors can improve the predictive performance for RARI in CC patients followed by radiotherapy.

## Highlights

• Predicting radiation-induced rectal injury is critical for cervical cancer prognosis.
• Brachytherapy applicator type was associated with acute rectal injury (P < 0.05).
• External area under curve was 0.773 for deep learning and 0.755 for dual-radiomics.
• Nomogram achieved best area under curve of 0.810 (internal) and 0.803 (external).

## Graphical abstract

**Figure:**
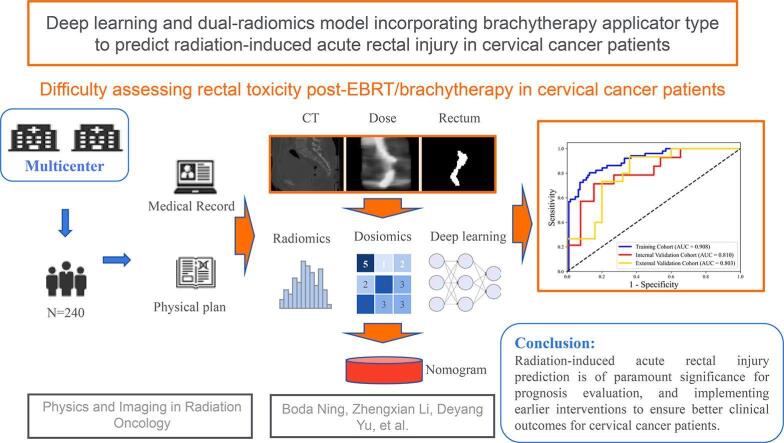

## 1 Introduction

Cervical cancer (CC) is the fourth most frequently diagnosed cancer and the fourth leading cause of cancer death in women [1]. Curative-intent treatment typically includes surgery, chemoradiotherapy (CRT), or their combination [2], [3]. In particular, the combination of external beam radiotherapy (EBRT) and brachytherapy (BT) is a standard treatment for CC patients. The EBRT part of the treatment aims to treat the whole pelvis, including the tumor and the lymph nodes at risk. The BT part of the treatment aims to boost the residual tumor in multiple fractions [4], [5]. However, radiotherapy increases complications, and radiation-induced rectal injury is among the most common [6]. Over 70% of patients receiving pelvic radiotherapy experience radiation-induced acute rectal injury (RARI), and 5–30% develop radiation-induced late rectal injury (RLRI) [7], [8]. Notably, the incidence of rectal toxicity following radiotherapy in CC patients ranges from approximately 26.1% to 38% [9], [10], [11]. Different dose regimens, fraction schemes, and BT techniques can lead to distinct toxicity outcomes [12]. These directly or indirectly affect treatment efficacy and prognosis [13], [14], [15]. Consequently, early detection and intervention of RARI are crucial for maximizing therapeutic effectiveness in CC patients.

Traditionally, many scoring systems, such as endoscopic scores and symptom scores, have been established to assess the severity of RARI [16]. On the other hand, although endoscopy is widely employed for RARI evaluation, it can easily cause mucosal bleeding, and biopsy may be prone to cause fistula formation [17]. In such cases, non-invasive computed tomography (CT) or magnetic resonance imaging (MRI) is a necessary supplement for better understanding RARI extent [18]. But these methods only enable post hoc intervention rather than proactive prediction. Therefore, there is an urgent need for a non-invasive, accurate diagnostic tool to effectively predict and manage RARI.

With the emergence of radiomics, numerous studies have demonstrated the predictive value of radiomic features for toxicity risk assessment [19], [20], [21], [22], [23]. Planning CT radiomic features, in particular, have shown potential for predicting radiation-induced rectal toxicities [24]. Additionally, The integration of dose-volume histogram (DVH) parameters and clinical factors with radiomic features has been demonstrated to improve the prediction accuracy of rectal toxicity in CC radiotherapy [25], [26]. Emerging evidence also suggests that convolutional neural network (CNN) models using dosiomic inputs may be more predictive than handcrafted dosiomic features [27]. Deep learning (DL) approaches for RARI prediction in CC remain underexplored, and few frameworks jointly model exposure from both EBRT and BT with rigorous external validation. Clinically, early identification of high-risk patients may enable adaptive strategies, such as adjusting EBRT fields and dose constraints, optimizing BT dwell times, or selecting less invasive applicators, to individualize rectal sparing while maintaining tumor control. Therefore, the purpose of this study is to analyze and compare the predictive performance of radiomics, dosiomics, and DL models, while developing a combined hybrid features to improve RARI prediction performance for CC patients before EBRT and BT, with its accuracy validated using external data.

## 2 Materials and methods

### 2.1 Patients and RARI grading

**Fig. 1:**
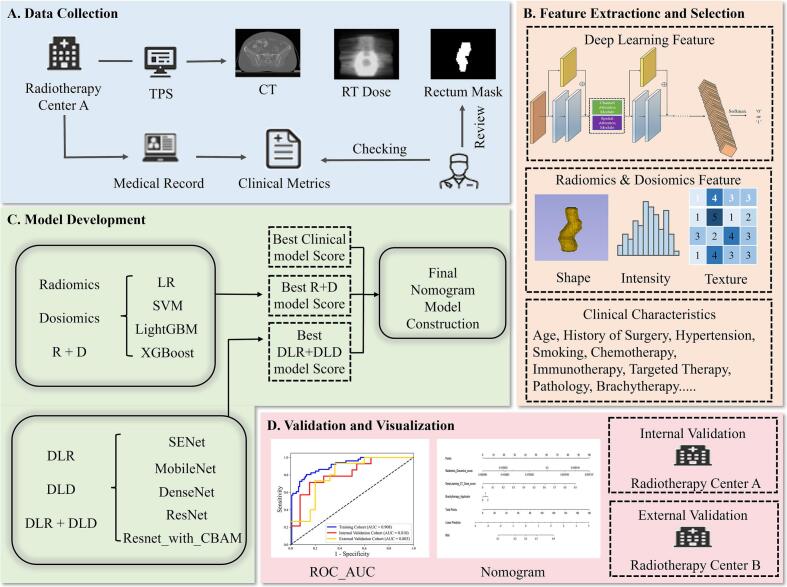
Analysis flowchart of each step in this study.

This retrospective multicenter study was approved by the institutional review boards of the participating centers, and informed consent was waived. Based on the exclusion criteria (Supplementary Fig. A.1), a total of 200 CC patients treated at Hospital A between 2022 and 2024 were enrolled and randomly split into training and internal validation cohorts (8:2). In addition, 40 CC patients treated at Hospital B between 2021 and 2023 constituted an external validation cohort. The study flowchart is shown in Fig. 1.

Adverse symptoms were recorded throughout treatment. After completion, patients underwent CT follow-up monthly during the first year and every 3 months thereafter up to 2 years. The diagnosis of RARI was made by a multidisciplinary oncologic team consisting of at least one radiation oncologist, one radiologist, and one gynecologist according to Common Terminology Criteria for Adverse Events (CTCAE) v5.0 in Supplementary Material Table B.1 [28]. The primary endpoint was RARI, defined a priori as CTCAE grade ≥ 2 occurring from the first fraction through 90 days after radiotherapy; grade ≥ 2 required medical intervention or limited instrumental activities of daily living, whereas grade < 2 was classified as Non-RARI.

### 2.2 Clinical treatment and image acquisition

EBRT was delivered using volumetric modulated arc therapy (VMAT) and TomoTherapy (Tomo) at Hospital A, with prescriptions of 45 Gy in 25 fractions or 50.4 Gy in 28 fractions [29]. Treatment planning and dose calculation followed center-specific treatment planning system (TPS) configurations [30]. BT differed by center: Hospital A used conventional 2D point-A prescription, whereas Hospital B used 3D CT-guided volumetric planning. Target volumes and organs at risk (OARs) were delineated according to Radiation Therapy and Oncology Group guidelines using AccuContour (V3.2; Manteia Technologies Co., Ltd.). Detailed treatment information is provided in Supplementary Material Doc C.1. CT images, dose distributions, and structures were exported from TPS at the original resolution of 512 × 512 × slice, and all data used for feature extraction were derived from EBRT. Clinical variables and DVH metrics were collected, including age, diabetes, hypertension, surgical history, BT details, rectal mean and maximum dose, and V_30Gy_, etc.

### 2.3 Radiomic and dosiomic features extraction and modeling

Radiomic features were extracted from the rectum on planning CT using PyRadiomics, and dosiomic features were extracted from the rectum on dose distributions using the same workflow [31]. To standardize feature computation, all CT images and dose distributions from both centers were resampled to 1 × 1 × 1 mm^3^ with B-spline interpolation. In total, 1288 radiomic features and 1288 dosiomic features were derived, covering shape, intensity, and texture descriptors [32]. The detailed information of radiomic and dosiomic features was presented in Supplementary Material Table D.1. All feature extraction algorithms were implemented adhering to the protocols set out by Image Biomarker Standardization Initiative [33].

To avoid overfitting, the Mann-Whitney *U* test was first used to select potentially informative features for RARI prediction (p < 0.05). Then, the least absolute shrinkage selection operator (LASSO) method was applied to screen the most significant features by tuning the regulation weight λ to achieve a maximum area under the curve (AUC) of receiver operating characteristic (ROC) curves. Four distinct machine learning (ML) classifiers were applied to build: (i) radiomics models, (ii) dosiomics models, and (iii) hybrid models incorporating both radiomic and dosiomic features. The details of model construction could be found in Supplementary Material Doc E.1.

### 2.4 Deep learning modeling

All images were cropped to 180 × 180 × 40 voxels, centered at the rectal coordinate midpoint, to standardize input size, ensure consistent region of interest (ROI) coverage, and reduce computational burden. Eleven DL networks were evaluated for RARI prediction, including SENet, Swin-Transformer, MobileNet series, DenseNet series, ResNet series, ResNet with convolutional block attention module (CBAM) [34], [35], [36], [37], [38], [39]. The detailed information of DL models was shown in Supplementary Fig. E.1. Models were trained under three input configurations: (i) CT images and rectum binary mask radiomics based on DL models (DLR), (ii) dose distribution and rectum binary mask dosiomics based on DL models (DLD), and (iii) combined CT, dose distribution and rectum binary mask based on DL models (DLR + DLD).

The DL models were implemented in Python 3.7 with TensorFlow 2.4.0 using an NVIDIA GeForce RTX 3090 GPU (24 GB). Each model underwent up to 200 epochs of training with early stopping (patience = 10), retaining the optimal weights when validation loss plateaued. Adam optimizer (initial learning rate 0.001) and binary cross-entropy were used as the loss function; batch size was set to 4 due to GPU memory limitations. The full code used in this work is openly available at GitHub (https://github.com/Rick-Ds/nbd_RRIPrediction_code).

### 2.5 Nomogram

DL features were extracted from a convolutional feature extractor whose architecture and hyperparameters were selected within the training cohort only, and then were combined with radiomic, dosiomic and clinical features to construct the nomogram. Clinical predictors of RARI were identified using univariate analysis and multivariate logistic regression in the training cohort. A multivariable logistic regression model was then fitted to integrate radiomics, dosiomics, DL, and clinical factors. Nomogram performance was evaluated in the training, internal validation, and external validation cohorts, with calibration assessed by the Hosmer-Lemeshow (H-L) test. Decision curve analysis (DCA) in the internal and external cohorts was used to quantify clinical utility.

### 2.6 Statistical analysis

Statistical analysis was conducted using SPSS software (version 22.0). Clinical variables between the RARI and Non-RARI groups were compared using Fisher exact test or chi-square test for categorical variables and Mann-Whitney *U* test or independent-sample *t*-test for continuous variables, as appropriate. A two-tailed p value < 0.05 was defined as statistical significance. In addition to ROC curves and AUC, accuracy, sensitivity, and specificity were reported to evaluate model discriminative capacity.

## 3 Results

A total of 200 CC patients from hospital A were randomly allocated to the training cohort (160) and internal validation cohort (40), with an additional 40 patients from hospital B serving as the external validation cohort. The RARI cases numbered 51 (31.9%), 14 (35%), and 15 (37.5%) in the training, internal validation, and external validation cohorts, respectively. There was a significant difference in hypertension and immunotherapy, targeted therapy and radiotherapy technique, volume of rectum D2cc between Non-RARI and RARI groups in the training, internal validation, and external validation cohorts, respectively. The characteristics of patients are shown in Table 1. Clinical model achieved an AUC of 0.455 and 0.513 in internal and external validation cohorts in Table 2, respectively.

**Table 1:** Patient characteristics in training cohort, internal validation cohort and external validation cohort.

Characteristics	Training Cohort (n = 160)			Internal Validation Cohort (n = 40)			External Validation Cohort (n = 40)		
	Non-RARI (n = 109)	RARI (n = 51)	P-Value	Non-RARI (n = 26)	RARI (n = 14)	P-Value	Non-RARI (n = 25)	RARI (n = 15)	P-Value
Age (years), Mean ± SD	56.0 ± 10.5	55.7 ± 9.9	0.887	55.2 ± 13.2	53.8 ± 10.7	0.727	58.9 ± 10.4	65.1 ± 8.3	0.058
Diabetes			0.384			1.000			0.705
Yes	3 (2.8%)	3 (5.9%)		1 (3.9%)	1 (7.1%)		5 (20.0%)	4 (26.7%)	
No	106 (97.2%)	48 (94.1%)		25 (96.1%)	13 (92.9%)		20 (80.0%)	11 (73.3%)	
Hypertension			0.039*			0.399			1.000
Yes	12 (11.0%)	12 (23.5%)		5 (19.2%)	1 (7.1%)		1 (4.0%)	1 (6.7%)	
No	97 (89.0%)	39 (76.5%)		21 (80.8%)	13 (92.9%)		24 (96.0%)	14 (93.3%)	
Surgery history			0.888			1.000			1.000
Yes	76 (69.7%)	35 (68.6%)		21 (80.8%)	11 (78.6%)		1 (4.0%)	0	
No	33 (30.3%)	16 (31.4%)		5 (19.2%)	3 (21.4%)		24 (96.0%)	15 (100.0%)	
Treatment target			0.593			0.864			1.000
Definitive	79 (72.5%)	39 (76.5%)		16 (61.5%)	9 (64.3%)		25 (100.0%)	15 (100.0%)	
postoperative	30 (27.5%)	12 (23.5%)		10 (38.5%)	5 (35.7%)		0	0	
Targeted therapy			0.720			0.043*			1.000
Yes	7 (6.4%)	2 (3.9%)		1 (3.9%)	4 (28.6%)		1 (4.0%)	0	
No	102 (93.6%)	49 (96.1%)		25 (96.2%)	10 (71.4%)		24 (96.0%)	15 (100.0%)	
Immunotherapy			0.038*			1.000			1.000
Yes	3 (2.8%)	4 (7.8%)		2 (7.7%)	1 (7.1%)		0	1 (6.7%)	
No	106 (97.3%)	47 (92.2%)		24 (92.3%)	13 (92.9%)		25 (100.0%)	14 (93.3%)	
Pathological type			0.454			0.687			0.519
SCC	89 (81.7%)	37 (72.6%)		20 (77.0%)	12 (85.7%)		23 (92.0%)	15 (100.0%)	
AC	17 (15.6)	12 (23.5%)		3 (11.5%)	2 (14.3%)		2 (8.0%)	0	
Other subtypes	3 (2.7%)	2 (3.9%)		3 (11.5%)	0		0	0	
Chemotherapy regimen			0.961			0.425			0.863
TP	54 (49.5%)	26 (51.0%)		11 (42.3%)	8 (57.2%)		1 (4.0%)	1 (6.7%)	
TC	30 (27.5%)	12 (23.5%)		10 (38.5%)	4 (28.6%)		0	0	
Single-agent platinum	12 (11.0%)	6 (11.8%)		2 (7.7%)	1 (7.1%)		17 (68.0%)	11 (73.3%)	
Other regimens	3 (2.8%)	1 (2.0%)		0	1 (7.1%)		0	0	
Without chemotherapy	10 (9.2%)	6 (11.7%)		3 (11.5%)	0		7 (28.0%)	3 (20.0%)	
CRT regimen			0.800			0.414			0.863
Concurrent CRT	58 (53.2%)	27 (53.0%)		10 (38.5%)	9 (64.3%)		17 (68.0%)	11 (73.3%)	
Sequential CRT	31 (28.4%)	12 (23.5%)		10 (38.5%)	4 (28.6%)		1 (4.0%)	1 (6.7%)	
Alternating CRT	10 (9.2%)	5 (9.8%)		3 (11.5%)	1 (7.1%)		0	0	
Radiotherapy alone	10 (9.2%)	7 (13.7%)		3 (11.5%)	0		7 (28.0%)	3 (20.0%)	
EBRT total dose (Gy), Mean ± SD	45.9 ± 2.0	45.7 ± 1.9	0.653	45.8 ± 2.2	45.8 ± 2.0	0.989	47.4 ± 2.1	48.3 ± 2.2	0.208
Rt_technique			0.171			< 0.001*			1.000
VMAT	42 (38.5%)	14 (27.5%)		15 (57.7%)	0		25 (100.0%)	15 (100.0%)	
Tomo	67 (61.5%)	37 (72.5%)		11 (42.3%)	14 (100.0%)		0	0	
SIB			0.635			0.393			0.107
Yes	25 (22.9%)	10 (19.6%)		6 (23.1%)	5 (35.7%)		17 (68.0%)	6 (40.0%)	
No	84 (77.1%)	41 (80.4%)		20 (76.9%)	9 (64.3%)		8 (32.0%)	9 (60.0%)	
PTV (cc), Mean ± SD	847.0 ± 440.2	851.5 ± 220.3	0.945	909.2 ± 213.9	820.4 ± 193.6	0.204	1450.7 ± 286.3	1590.0 ± 374.2	0.192
BT applicator			0.772			0.796			0.545
Fletcher Williamson applicator	25 (22.9%)	10 (19.6%)		4 (15.4%)	3 (21.4%)		24 (96.0%)	13 (86.7%)	
Vaginal applicator	80 (73.4%)	38 (74.5%)		21 (80.8%)	11 (78.6%)		1 (4.0%)	2 (13.3%)	
Without BT	4 (3.7%)	3 (5.9%)		1 (3.8%)	0		0	0	
BT total dose (Gy), Mean ± SD	21.9 ± 6.8	21.3 ± 7.8	0.669	21.7 ± 7.6	21.9 ± 5.1	0.942	28.4 ± 1.5	28.0 ± 0.0	0.149
DVH parameters									
Rectum_Dmax (Gy)	48.6 ± 2.8	48.1 ± 2.2	0.205	48.8 ± 1.9	48.0 ± 2.3	0.245	51.2 ± 2.5	52.3 ± 3.0	0.216
Rectum_Dmean (Gy)	28.8 ± 3.0	29.3 ± 2.9	0.293	28.5 ± 3.0	29.3 ± 2.3	0.407	40.4 ± 4.1	43.1 ± 4.2	0.055
Rectum_V30 (%)	45.0 ± 7.4	45.9 ± 7.6	0.490	45.5 ± 5.9	45.2 ± 4.1	0.863	83.9 ± 9.5	89.8 ± 9.4	0.063
Rectum_V40 (%)	21.7 ± 7.5	21.1 ± 8.9	0.695	22.3 ± 5.2	20.8 ± 5.0	0.394	66.1 ± 12.8	74.1 ± 13.2	0.067
Rectum_D2cc (Gy)	46.6 ± 2.8	46.0 ± 2.8	0.181	46.9 ± 2.0	45.9 ± 2.5	0.161	50.0 ± 2.7	50.6 ± 2.7	0.476
Rectum_V_D2cc (%)	2.8 ± 1.4	3.2 ± 1.8	0.221	2.6 ± 1.0	3.3 ± 2.7	0.215	4.3 ± 1.5	5.4 ± 2.0	0.044*

Note: All DVH parameters are EBRT-only. Abbreviations: SCC: Squamous cell carcinoma; AC: Adenocarcinoma; TP: paclitaxel+cisplatin; TC: paclitaxel + carboplatin; Single-Agent Platinum: cisplatin or carboplatin or nedaplatin; CRT: Chemoradiotherapy; EBRT: External beam radiotherapy; BT: Brachytherapy; Rt_technique: radiotherapy technique used to treat patient; VMAT: volumetric modulated arc therapy; SIB: simultaneous integrated boost intensity modulated radiotherapy; PTV: planning tumor volume; Rectum_Dmax: rectum Dmax; Rectum_Dmean: rectum Dmean; Rectum_V30: rectum V30 (%); Rectum_V40: rectum V40 (%); Rectum_D2cc: Rectum D2cc (cm^3^); Rectum_V_D2cc(%): volume of rectum D2cc; Chi-squared test and Fisher’s exact test for categorized variables; independent *t* test for continues variables. * The p value < 0.05 is considered statistically significant.

**Table 2:** The performance of radiomics, dosiomics, DL and clinical models with single input in the training, internal validation and external validation cohorts.

Input	Model	Training Cohort				Internal Validation Cohort				External Validation Cohort			
		AUC (95% CI)	ACC	Sen	Spe	AUC (95% CI)	ACC	Sen	Spe	AUC (95% CI)	ACC	Sen	Spe
R feature	LR	0.769 (0.692–0.845)	0.738	0.686	0.761	0.648 (0.472–0.824)	0.650	0.714	0.615	0.659 (0.483–0.834)	0.675	0.733	0.640
	SVM	0.748 (0.669–0.827)	0.675	0.596	0.843	0.670 (0.500–0.840)	0.575	0.346	1.000	0.624 (0.442–0.806)	0.650	0.600	0.733
	LightGBM	0.801 (0.728–0.874)	0.769	0.725	0.789	0.728 (0.569–0.887)	0.725	0.429	0.885	0.704 (0.524–0.866)	0.625	1.000	0.400
	XGBoost	0.852 (0.782–0.923)	0.838	0.706	0.899	0.740 (0.586–0.895)	0.625	1.000	0.423	0.728 (0.518–0.890)	0.750	0.533	0.880

D feature	LR	0.860 (0.799–0.920)	0.800	0.784	0.807	0.585 (0.345–0.756)	0.700	0.429	0.846	0.512 (0.322–0.702)	0.550	0.867	0.360
	SVM	0.750 (0.671–0.830)	0.688	0.606	0.863	0.687 (0.489–0.885)	0.775	0.500	0.923	0.560 (0.364–0.757)	0.625	0.560	0.733
	LightGBM	0.726 (0.644–0.808)	0.675	0.686	0.670	0.641 (0.468–0.815)	0.575	0.929	0.385	0.625 (0.447–0.803)	0.650	0.667	0.640
	XGBoost	0.787 (0.712–0.862)	0.725	0.804	0.688	0.670 (0.493–0.848)	0.675	0.643	0.692	0.704 (0.542–0.866)	0.650	1.000	0.440

DLR feature	SEnet	0.701 (0.622–0.780)	0.625	0.863	0.514	0.665 (0.490–0.835)	0.700	0.786	0.654	0.664 (0.457–0.853)	0.750	0.400	0.960
	Mobile-v3	0.754 (0.667–0.837)	0.706	0.686	0.716	0.670 (0.497–0.845)	0.650	0.857	0.538	0.627 (0.429–0.803)	0.675	0.600	0.720
	Densenet-201	0.788 (0.710–0.855)	0.731	0.765	0.716	0.750 (0.580–0.896)	0.750	0.857	0.692	0.675 (0.484–0.849)	0.625	0.867	0.480
	Resnet-34	0.752 (0.677–0.826)	0.637	0.863	0.532	0.703 (0.516–0.865)	0.625	0.786	0.538	0.680 (0.508–0.848)	0.650	0.800	0.560
	Resnet_with_CBAM	0.827 (0.766–0.881)	0.744	1.000	0.624	0.739 (0.574–0.875)	0.675	0.929	0.538	0.728 (0.518–0.890)	0.750	0.600	0.840

DLD feature	SEnet	0.764 (0.684–0.836)	0.725	0.647	0.761	0.681 (0.486–0.832)	0.675	0.786	0.615	0.619 (0.442–0.783)	0.600	0.933	0.400
	Mobile-v3	0.665 (0.575–0.753)	0.544	0.824	0.413	0.591 (0.403–0.784)	0.675	0.500	0.769	0.592 (0.396–0.790)	0.600	0.667	0.560
	Densenet-201	0.716 (0.628–0.796)	0.675	0.706	0.661	0.673 (0.487–0.840)	0.700	0.643	0.731	0.621 (0.429–0.805)	0.650	0.667	0.640
	Resnet-34	0.743 (0.661–0.816)	0.619	0.922	0.477	0.703 (0.536–0.850)	0.650	1.000	0.462	0.712 (0.519–0.871)	0.675	0.800	0.600
	Resnet_with_CBAM	0.774 (0.697–0.844)	0.713	0.961	0.596	0.728 (0.577–0.875)	0.750	0.857	0.692	0.685 (0.487–0.875)	0.725	0.600	0.800

Clinical	LR	0.525 (0.449–0.592)	0.412	0.804	0.229	0.455 (0.320–0.582)	0.650	0.901	0.143	0.513 (0.448–0.600)	0.625	0.912	0.106

Abbreviations: R feature: radiomics feature; D feature: dosiomics feature; DLR feature: radiomics based on deep learning feature; DLD feature: dosiomics based on deep learning feature; LR: logistic regression; SVM: support vector machine; LightGBM: light gradient boosting machine; XGBoost: extreme gradient boosting; AUC: area under the curve; ACC: accuracy; Sen: sensitivity; Spe: specificity.

**Fig. 2:**
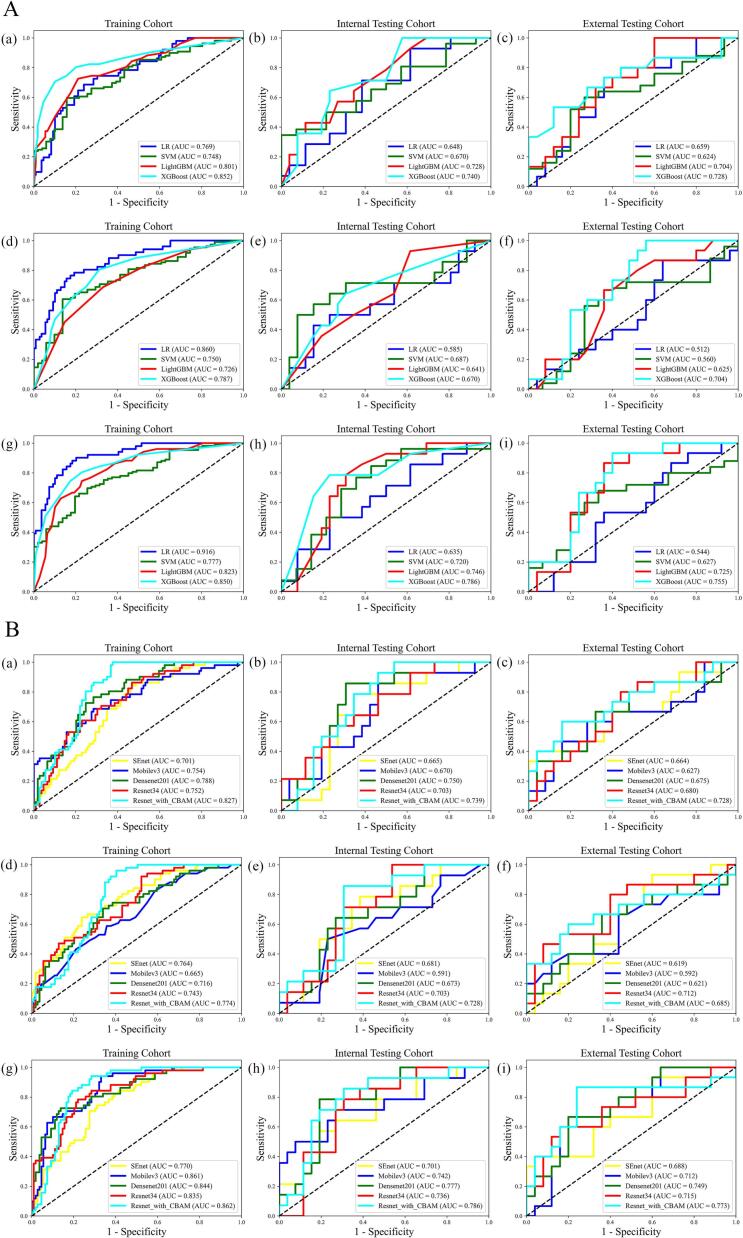
The ROC curves in the training cohort, internal validation cohort and external validation cohort for the different models. A (a, b, c) & A (d, e, f) The ROC curves for models with radiomic features and dosiomic features in the training cohort, internal validation cohort and external validation cohort, respectively. A (g, h, i) The ROC curves for models with radiomic combined dosiomic features in the training cohort, internal validation cohort and external validation cohort, respectively. B (a, b, c) & B (d, e, f) The ROC curves for models with single input of CT images and single input of dose distributions in the training cohort, internal validation cohort and external validation cohort, respectively. B (g, h, i) The ROC curves for models with CT images combined dose distributions in the training cohort, internal validation cohort and external validation cohort, respectively.

11 radiomic and 11 dosiomic features were selected after Mann–Whitney U testing and LASSO. The corresponding features and coefficients are reported in Supplementary Material Table F and Fig. F. Among the four ML models, XGBoost achieved the best radiomics model performance as shown in Fig. 2A(a)–(c) with AUCs of 0.852 (95% CI: 0.782–0.923), 0.740 (95% CI: 0.586–0.895), 0.728 (95% CI: 0.518–0.890) in training, internal validation, and external validation cohorts, respectively. For dosiomics models, XGBoost showed overall optimal performance as shown in Fig. 2A(d)–(f) with corresponding AUCs of 0.787 (95% CI: 0.712–0.862), 0.670 (95% CI: 0.493–0.848), 0.704 (95%CI: 0.542–0.866). The radiomics + dosiomics model further improved discrimination as shown in Fig. 2A(g)–(i), achieving AUCs of 0.850 (95% CI: 0.784–0.916), 0.786 (95% CI: 0.632–0.940) and 0.755 (95% CI: 0.603–0.906) across the three cohorts. Detailed comparison was shown in Table2. The visualization of feature importance based on XGBoost was shown in Supplementary Material Fig. G.

The performance of 11 DL models in the training, internal validation cohorts was summarized in Supplementary Material Table H.1. As shown in Table 2 and Fig. 2B(a)–(f), Resnet_with_CBAM achieved the best prediction performance with AUCs of 0.827 (95% CI: 0.766–0.881), 0.739 (95% CI: 0.574–0.875), 0.728 (95% CI: 0.518–0.890) and 0.774 (95% CI: 0.697–0.844), 0.728 (95% CI: 0.577–0.875), 0.685 (95% CI: 0.487–0.875) for the input of DLR or DLD in training, internal and external validation cohorts, respectively. As shown in Fig. 2B(g)–(i), with combined input (DLR + DLD), Resnet_with_CBAM achieved the best AUCs of 0.862 (95% CI: 0.803–0.918), 0.786 (95% CI: 0.615–0.926) and 0.773 (95% CI: 0.583–0.925) across the three cohorts. Grad-CAM was used to visualize regions contributing most to prediction, as shown in Supplementary Material Fig. G.3. Detailed performance of hybrid models were shown in Table 3.

**Table 3:** The performance of models with combined different inputs in the training, internal validation and external validation cohorts.

Input	Model	Training Cohort				Internal Validation Cohort				External Validation Cohort			
		AUC (95% CI)	ACC	Sen	Spe	AUC (95% CI)	ACC	Sen	Spe	AUC (95% CI)	ACC	Sen	Spe
R + D feature	LR	0.916 (0.873–0.960)	0.831	0.902	0.798	0.635 (0.451–0.818)	0.675	0.500	0.769	0.544 (0.361–0.728)	0.525	0.867	0.320
	SVM	0.777 (0.703–0.850)	0.694	0.642	0.804	0.720 (0.535–0.905)	0.750	0.846	0.571	0.627 (0.447–0.807)	0.675	0.680	0.667
	LightGBM	0.823 (0.755–0.890)	0.762	0.745	0.771	0.746 (0.592–0.900)	0.725	0.786	0.692	0.725 (0.565–0.886)	0.725	0.867	0.640
	XGBoost	0.850 (0.784–0.916)	0.781	0.804	0.771	0.786 (0.632–0.940)	0.775	0.786	0.769	0.755 (0.603–0.906)	0.725	0.933	0.600

DLR + DLD feature	SEnet	0.770 (0.693–0.843)	0.706	0.745	0.688	0.701 (0.520–0.859)	0.725	0.571	0.808	0.688 (0.500–0.846)	0.750	0.400	0.960
	Mobile-v3	0.861 (0.798–0.914)	0.756	0.941	0.670	0.742 (0.556–0.900)	0.725	0.714	0.731	0.712 (0.544–0.872)	0.750	0.600	0.840
	Densenet-201	0.844 (0.773–0.905)	0.819	0.725	0.862	0.777 (0.624–0.918)	0.800	0.786	0.808	0.749 (0.578–0.887)	0.750	0.667	0.800
	Resnet-34	0.835 (0.768–0.897)	0.781	0.784	0.780	0.736 (0.568–0.875)	0.725	0.786	0.692	0.715 (0.538–0.878)	0.750	0.600	0.840
	Resnet_with_CBAM	0.862 (0.803–0.918)	0.787	0.941	0.716	0.786 (0.615–0.926)	0.750	0.857	0.692	0.773 (0.583–0.925)	0.800	0.867	0.760

R + D + DLR + DLD + Clinical	Nomogram	0.908 (0.855–0.951)	0.850	0.804	0.872	0.810 (0.652–0.946)	0.800	0.714	0.846	0.803 (0.651–0.922)	0.750	0.933	0.640

Abbreviations: R feature: radiomics feature; D feature: dosiomics feature; DLR feature: radiomics based on deep learning feature; DLD feature: dosiomics based on deep learning feature; LR: logistic regression; SVM: support vector machine; LightGBM: light gradient boosting machine; XGBoost: extreme gradient boosting; AUC: area under the curve; ACC: accuracy; Sen: sensitivity; Spe: specificity

**Fig. 3:**
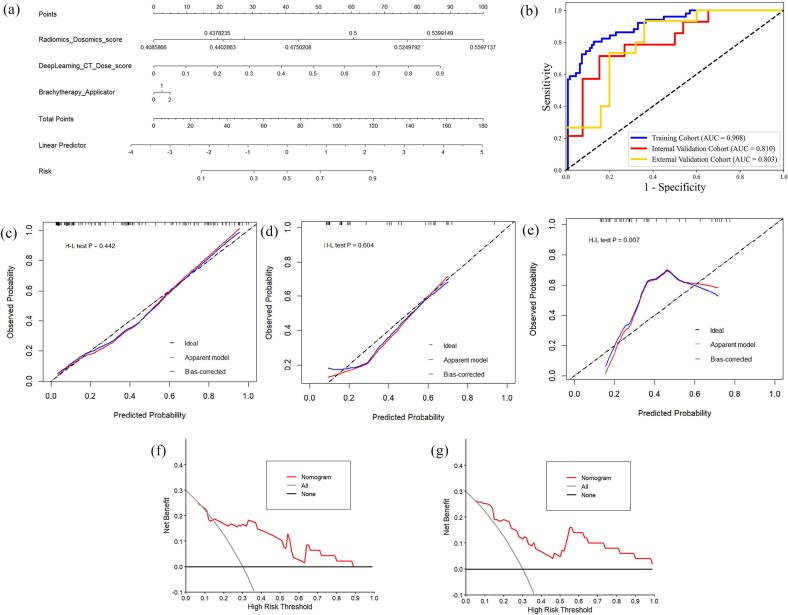
Performance evaluation of the nomogram. (a) Nomogram construction; (b) ROC curve of nomogram in the training cohort, internal validation cohort and external validation cohort; (c)-(e) Calibration curves of nomogram in the training cohort, internal validation cohort and external validation cohort; (f) Decision curve analysis for the internal validation cohort; (g) Decision curve analysis for the external validation cohort.

According to the univariate and multivariate analysis of clinical parameters (Supplementary Material Table I.1), BT applicator (p < 0.05) was independent predictors of RARI. A nomogram integrating DL, radiomic and dosiomic features with independent clinical risk factor was presented in Fig. 3(a). The performance of the nomogram in the training, internal validation and external validation cohorts was shown in Fig. 3(b) with an AUC of 0.908 (95% CI: 0.855–0.951), 0.810 (95% CI: 0.652–0.946) and 0.803 (95% CI: 0.651–0.922), respectively. The calibration curve of the bootstrap resampling-validated nomogram in different cohorts was shown in Fig. 3(c)–(e), which demonstrated a good agreement between the predicted probabilities of RARI and the true observed probabilities. The decision curve analysis (DCA) for the nomogram in the internal validation and external validation cohorts was shown in Fig. 3(f), (g).

## 4 Discussion

This study systematically evaluated the predictive performance of DL models, dual-radiomics models, and their integrated approach for RARI. The radiomics + dosiomics model achieved AUCs of 0.786 and 0.755 in the internal and external validation cohorts, respectively, while the DLR + DLD model achieved AUCs of 0.786 and 0.773. The nomogram integrating DL, radiomics, dosiomics, and clinical factors demonstrated the best performance, with AUCs of 0.810 and 0.803 in the internal and external validation cohorts, respectively.

With combined EBRT and BT irradiation, some increase in rectal dose is often unavoidable. In our cohort, BT applicator type was significantly associated with RARI (p = 0.033), a factor seldom emphasized previously, and may capture technique- and geometry-driven differences in rectal dose exposure [40]. However, the RARI rates were similar for Fletcher-Williamson versus vaginal applicators (32.9% vs 33.3%), and the subgroup without BT was small, which could not support the conclusion that a specific applicator is intrinsically riskier; the association more likely reflects case mix and BT practice. Prior studies have implicated D2cc and vascular comorbidities (hypertension, diabetes, atherosclerosis) as risk factors for rectal injury [14], [26], [41]. In line with these reports, immunotherapy, hypertension, and rectal V_D2cc showed borderline associations in multivariable analysis (0.05 < p < 0.1). Overall, RARI incidence was 32.5% (65/200), compared with 22.5% and 50.0% reported by Wei et al. and Xue et al., respectively [25], [26]. Such variability is plausibly attributable to differences in radiotherapy techniques and BT schemes across cohorts.

Radiomics and dosiomics quantify high-dimensional descriptors from anatomy and dose distributions and have been increasingly explored for rectal toxicity prediction. In prostate cancer, Hossein et al. improved rectal toxicity prediction by integrating MRI radiomics with clinical and DVH metrics, achieving an AUC of 0.79 [42]. In this study, the XGBoost model based solely on radiomic or dosiomic features achieved AUCs of 0.740, 0.728 and 0.670, 0.704, while combining radiomics and dosiomics achieved an AUC of 0.786, 0.755 in internal validation and external validation cohorts, respectively. These findings support the feasibility of integrating CT radiomics and rectal dosiomics for RARI prediction in CC. The most informative CT radiomic features were predominantly first-order intensity and texture-heterogeneity descriptors, whereas dosiomic features captured high-dose hotspots and steep gradients, suggesting that baseline structural heterogeneity and spatially inhomogeneous high-dose exposure of the rectal wall jointly contribute to RARI risk, consistent with prior work [24], [25], [26]. Several methodological directions may further strengthen radiomics- and dosiomics-based prediction. Adachi et al. proposed dose-segmented dosiomics to better capture dose-level–specific morphologic patterns, improving radiation pneumonia prediction [43]. In addition, MRI offers superior soft-tissue contrast for rectal wall characterization. Xue et al. demonstrated MRI delta radiomics for radiation proctitis severity prediction in CC [26], and Wei et al. reported benefits of multi-sequence MRI radiomics [44]. Multimodal integration has also been advocated in gynecologic oncology [45], motivating future studies incorporating standardized MRI acquisition and harmonized multimodal features.

Compared with handcrafted features, DL enables end-to-end representation learning and can capture spatial patterns that are difficult to predefine. Although DL studies focused on rectal toxicity after pelvic radiotherapy remain limited, DL has shown advantages in related tasks such as radiation pneumonitis [46], [47]. Elhaminia et al. developed a multimodal DL fusion model using CT, dose, and clinical data to predict bowel urgency toxicity, reporting an AUC up to 0.88 [48]. In our experiments, most of the 11 DL networks performed robustly. ResNet_with_CBAM achieved the best AUCs among DLR and DLD models, comparable to radiomics and dosiomics models. With combined inputs of CT, dose distribution, and rectum mask, 10 of 11 models reached AUC > 0.7 in internal validation, again led by ResNet_with_CBAM. Grad-CAM highlighted that model focused on the rectal wall and adjacent tissues within high-dose and steep-gradient regions. Together, these results support DL as a feasible and robust approach for RARI prediction and suggest added value from integrating engineered radiomic/dosiomic information.

To facilitate individualized risk communication and clinical use, we developed a nomogram integrating radiomics, dosiomics, DL features, and BT applicator information, which achieved the best discrimination [49], [50]. Calibration showed good internal agreement (H-L, p = 0.604) but significant external deviation (H-L, p = 0.007), plausibly reflecting between-hospital differences in case mix, clinical practice, and predictor measurement; such dataset shift can impair calibration even when discrimination remains acceptable [51]. The DCA further indicated positive net benefit across relevant threshold probabilities, supporting potential clinical utility. The nomogram is intended for pre-treatment or plan-time use: once contours and plans are finalized, features can be automatically extracted to generate an individualized RARI risk estimate. An a priori action threshold within the DCA-positive range can then trigger low-harm mitigation, including stricter rectal constraints with EBRT re-optimization, careful review of BT technique and rectal dose indices, and enhanced on-treatment monitoring. Threshold selection may favor sensitivity for low-risk triage, whereas resource-intensive adaptations may require higher specificity depending on clinical priorities.

This study has several limitations. First, all CC patients from Hospital A underwent 2D BT rather than 3D, preventing reconstruction of BT dose in a consistent voxel-wise format. Consequently, we could not use the equivalent dose in 2-Gy fractions (EQD2) method to superimpose EBRT and BT dose distributions or accurately quantify cumulative rectal dose from both modalities. Future work will prioritize cohorts treated with 3D image-guided BT to enable EBRT + BT cumulative dose modeling. Second, although 11 DL networks were evaluated, most were CNN-based; non-CNN architectures, including diffusion models, may further improve performance. Third, the external validation cohort was limited, yielding wide confidence intervals, and overfitting cannot be fully excluded given the high-dimensional feature space. In addition, Only clinician-reported acute RARI within available follow-up was analyzed; late toxicity was not assessed and analyzed due to lacking long-term data. Finally, inputs were restricted to planning CT. Standardized MRI with deformable CT-MR registration, harmonization, and multimodal feature extraction may improve prediction in future work.

In conclusion, this study successfully developed a nomogram integrating radiomic features, dosiomic features, DL features, and clinical factors to improve the predictive performance for RARI in CC patients undergoing radiotherapy. These findings could guide clinicians to proactively optimize treatment plans for patients predicted to be at risk of RARI, thereby improving quality of life and clinical outcomes.

## CRediT authorship contribution statement

**Boda Ning:** Software, Data curation, Writing – original draft, Validation, Formal analysis, Investigation. **Zhengxian Li:** Data curation, Formal analysis, Writing – review & editing. **Deyang Yu:** Resources, Data curation, Conceptualization, Funding acquisition. **Chenyu Li:** Data curation, Software. **Qi Liu:** Formal analysis, Funding acquisition. **Yanling Bai:** Conceptualization, Methodology, Writing – review & editing, Supervision, Project administration, Funding acquisition.

## Declaration of competing interest

The authors declare that they have no known competing financial interests or personal relationships that could have appeared to influence the work reported in this paper.
