# Supplementary material for: Deep learning and dual-radiomics model incorporating brachytherapy applicator type to predict radiation-induced acute rectal injury in cervical cancer patients
Source: Phys Imaging Radiat Oncol. 2026 Jan 20;37:100908. doi: 10.1016/j.phro.2026.100908 (PMC12861146; doi:10.1016/j.phro.2026.100908)
Supplement: Supplementary Data 1 [file mmc1.pdf]

Supplementary Material

A. Exclusion criteria of patients

B. RARI assessment

C. Technical details of treatment planning and brachytherapy

D. Radiomics and dosiomics features extraction

E. The details of model construction

F. The details of the selected radiomics features and dosiomics features

G. The visualization of feature importance

H. The performance of 11 deep learning models

I. Univariate and multivariate analysis of clinical factors in predicting RARI

A. Exclusion criteria of CC patients

Figure A.1 Exclusion criteria of CC patients

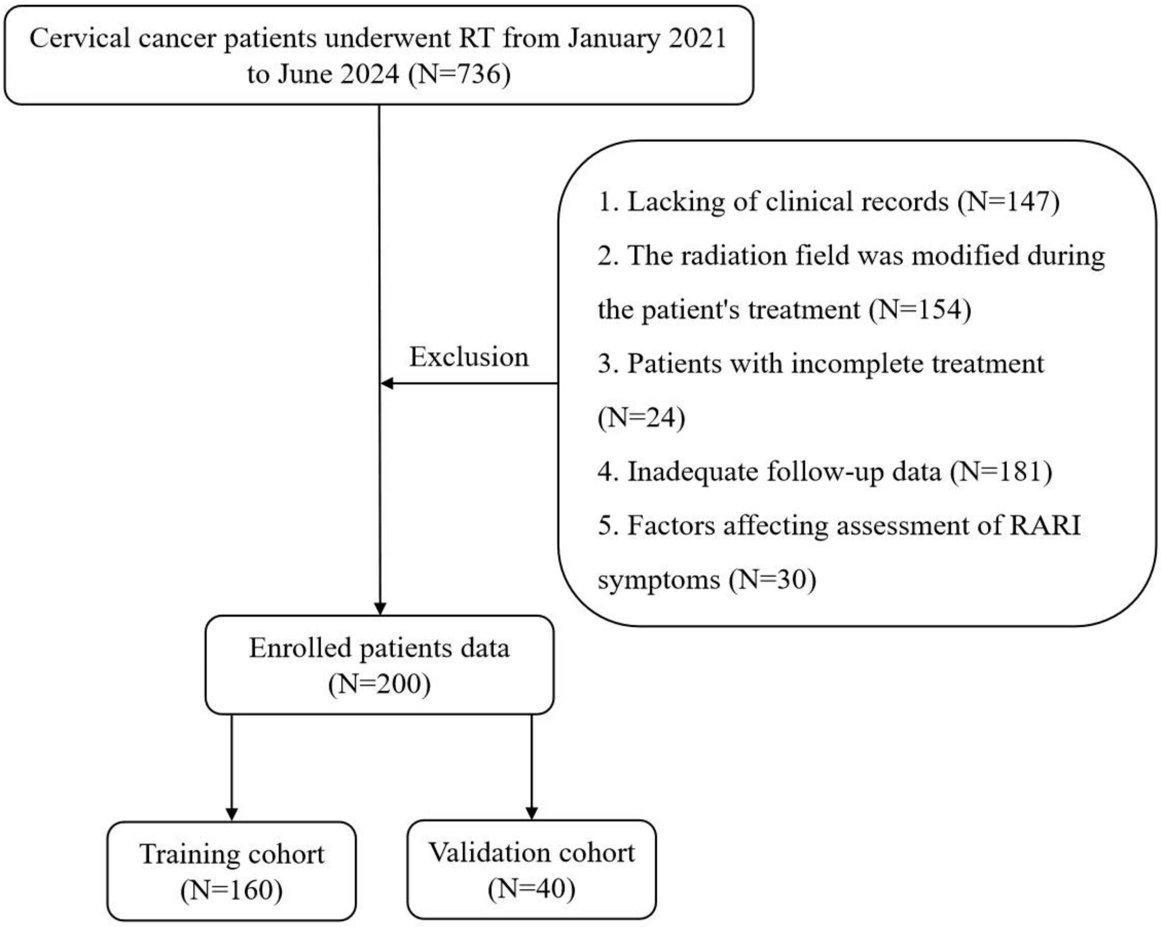

B. RARI assessment

Table B.1 Common Terminology Criteria for Adverse Events (CTACE) v5.0 for RARI.

Grade	Common Terminology Criteria for Adverse Events (CTACE) v5.0 for RARI
0	None
1	Asymptomatic; clinical or diagnostic observations only; intervention not indicated
2	Symptomatic (e.g., rectal pain, mucus discharge, or tenesmus); medical intervention indicated; limiting instrumental ADL*
3	Severe symptoms (e.g., bleeding or severe pain); invasive intervention indicated (e.g., cauterization, stent placement); limiting self-care ADL**
4	Life-threatening consequences; urgent operative intervention indicated
5	Death

C. Technical details of treatment planning and brachytherapy

Doc C.1 Technical details of treatment planning and brachytherapy.

RT planning CT images were acquired on a Philips Brilliance Big Bore CT scanner at a slice thickness of 5 mm. At Hospital A, VMAT plans were generated using the Pinnacle treatment planning system (clinical version 16.2) with a 4-mm dose grid and a collapsed cone convolution dose calculation algorithm, and Tomo plans were generated using the Tomo-HD station (clinical version 5.1.9.2) with normal dose grid and Convolution/Superposition dose calculation algorithm. At Hospital B, VMAT plans were generated using the Monaco TPS (clinical version 5.11) with a 3-mm dose grid and a fast Monte Carlo dose calculation algorithm. All VMAT and Tomo plans were delivered on Elekta Synergy, Infinity, VersaHD linac (Elekta, Stockholm, Sweden) and TomoTherapy (Sunnyvale, CA, USA) with a 6 MV X-ray. Hospital A employed a conventional two-dimensional (2D) BT system (utilizing point-A prescription) using Flexitron (Elekta, Stockholm, Sweden) with the radiation source (Iridium-192), whereas hospital B utilized a three-dimensional (3D) image-guided BT system (utilizing CT-based volumetric planning) using MicroSelectron V3 (Elekta, Stockholm, Sweden), also equipped with an Iridium-192 source. The source length typically ranged between 2 – 10 cm, and the prescription dose varied from 12-36 Gy in 4-7 Gy/fraction. The applicators that all BT used were Fletcher Williamson applicator or Vaginal applicator.

D. Radiomics and dosiomics features extraction

The same radiomics features and dosiomics features were extracted by Pyradiomics. Features are shown in the table below.

Table D.1 List of radiomics and dosiomics features extracted in this study.

Feature class	Features	Number of features
Firstorder	10Percentile,90Percentile,Energy,Entropy,InterquartileRange,Kurtosis,Maximum,MeanAbsoluteDeviation,Mean,Median,Minimum,Range,RobustMeanAbsoluteDeviation,RootMeanSquared,Skewness,TotalEnergy,Uniformity,Variance	18
Shape	Elongation,Flatness,LeastAxisLength,MajorAxisLength,Maximum2DDiameter(Column),Maximum2DDiameter(Row),Maximum2DDiameterSlice,Maximum3DDiameter,MeshVolume,MinorAxisLength,Sphericity,SurfaceArea,SurfaceVolumeRatio, VoxelVolume	14
GLCM	Autocorrelation,JointAverage,ClusterProminence,ClusterShade,ClusterTendency,Contrast,Correlation,DifferenceAverage,DifferenceEntropy,DifferenceVariance,JointEnergy, JointEntropy, Imc1, Imc2, Idm, Idmn, Id, Idn, InverseVariance, MaximumProbability, SumEntropy, SumSquares	22
GLSZM	GrayLevelNonUniformity,GrayLevelNonUniformityNormalized,GrayLevelVariance,HighGrayLevelZoneEmphasis,LargeAreaEmphasis,LargeAreaHighGrayLevelEmphasis,LargeAreaLowGrayLevelEmphasis,LowGrayLevelZoneEmphasis,SizeZoneNonUniformity,SizeZoneNonUniformityNormalized,SmallAreaEmphasis,SmallAreaHighGrayLevelEmphasis,SmallAreaLowGrayLevelEmphasis,ZoneEntropy,ZonePercentage,ZoneVariance	16
GLRLM	GrayLevelNonUniformity,GrayLevelNonUniformityNormalized,GrayLevelVariance,HighGrayLevelRunEmphasis,LongRunEmphasis,LongRunHighGrayLevelEmphasis,LongRunLowGrayLevelEmphasis,LowGrayLevelRunEmphasis,RunEntropy,RunLengthNonUniformity,RunLengthNonUniformityNormalized,RunPercentage,RunVariance,ShortRunEmphasis,ShortRunHighGrayLevelEmphasis, ShortRunLowGrayLevelEmphasis	16

NGTDM	Busyness,Coarseness,Complexity,Contrast,Strength,	5
GLDM	DependenceEntropy,DependenceNonUniformity,DependenceNonUniformityNormalized,DependenceVariance,GrayLevelNonUniformity,GrayLevelVariance,HighGrayLevelEmphasis,LargeDependenceEmphasis,LargeDependenceHighGrayLevelEmphasis,LargeDependenceLowGrayLevelEmphasis,LowGrayLevelEmphasis,SmallDependenceEmphasis,SmallDependenceHighGrayLevelEmphasis,SmallDependenceLowGrayLevelEmphasis	14

GLCM, gray-level co-occurrence matrix; GLRLM, gray-level run-length matrix; GLSZM, gray-level size zone matrix; NGTDM, neighborhood gray-tone difference matrix.

Note: it's worth noting that the names in this list are different from IBSI names, but the methods are the same. The algorithm details about feature extraction can be referred to: https://pyradiomics.readthedocs.io

Wavelet features were acquired using Coif wavelet on the basis of eight wavelet decompositions from three-dimensional sequences. All possible combinations of applying either a High or a Low pass filter include LLH, LHL, LHH, HLL, HLH, HHL, HHH and LLL. Therefore, 728 wavelet features were extracted.

The mathematical definitions are given on the Pyradiomics feature documentation (https://pyradiomics.readthedocs.io/en/latest/features.html). Most of them are described by the Imaging Biomarker Standardization Initiative (IBSI), which are available in a separate document by Zwanenburg et al (2016).

E. The details of model construction

Doc E.1 Radiomics and dosomics models construction.

Four machine learning (ML) models with the input of radiomics features: logistic regression (LR) (family = binomial(link = "logit"), support vector machine (SVM) (type = 'C', kernel = “radial”, gamma = 0.000008, and cost = 0.00001), Light Gradient Boosting Machine (LightGBM) (learning_rate = 0.68, num_leaves = 31, max_depth = 20, min_data_in_leaf = 20, min_sum_hessian_in_leaf = 1e-3, lambda = 0.01, gamma = 0.0, feature_fraction = 0.9,m bagging_fraction = 0.8, bagging_freq = 5) and extreme gradient boosting (XGboost) (eta = 0.03, max_depth = 10, subsample = 0.67, and colsample_bytree = 0.85).

Four machine learning (ML) models with the input of dosomics features: logistic regression (LR) (family = binomial(link = "logit"), support vector machine (SVM) (type = 'C', kernel = “radial”, gamma = 0.0001, and cost = 10), Light Gradient Boosting Machine (LightGBM) (learning_rate = 0.5, num_leaves = 31, max_depth = 20, min_data_in_leaf = 20, min_sum_hessian_in_leaf = 1e-3, lambda = 0.01, gamma = 0.0, feature_fraction = 0.9,m bagging_fraction = 0.8, bagging_freq = 5) and extreme gradient boosting (XGboost) (eta = 0.1, max_depth = 10, subsample = 0.65, and colsample_bytree = 0.83).

Figure E.1 Resnet_with_CBAM model construction.

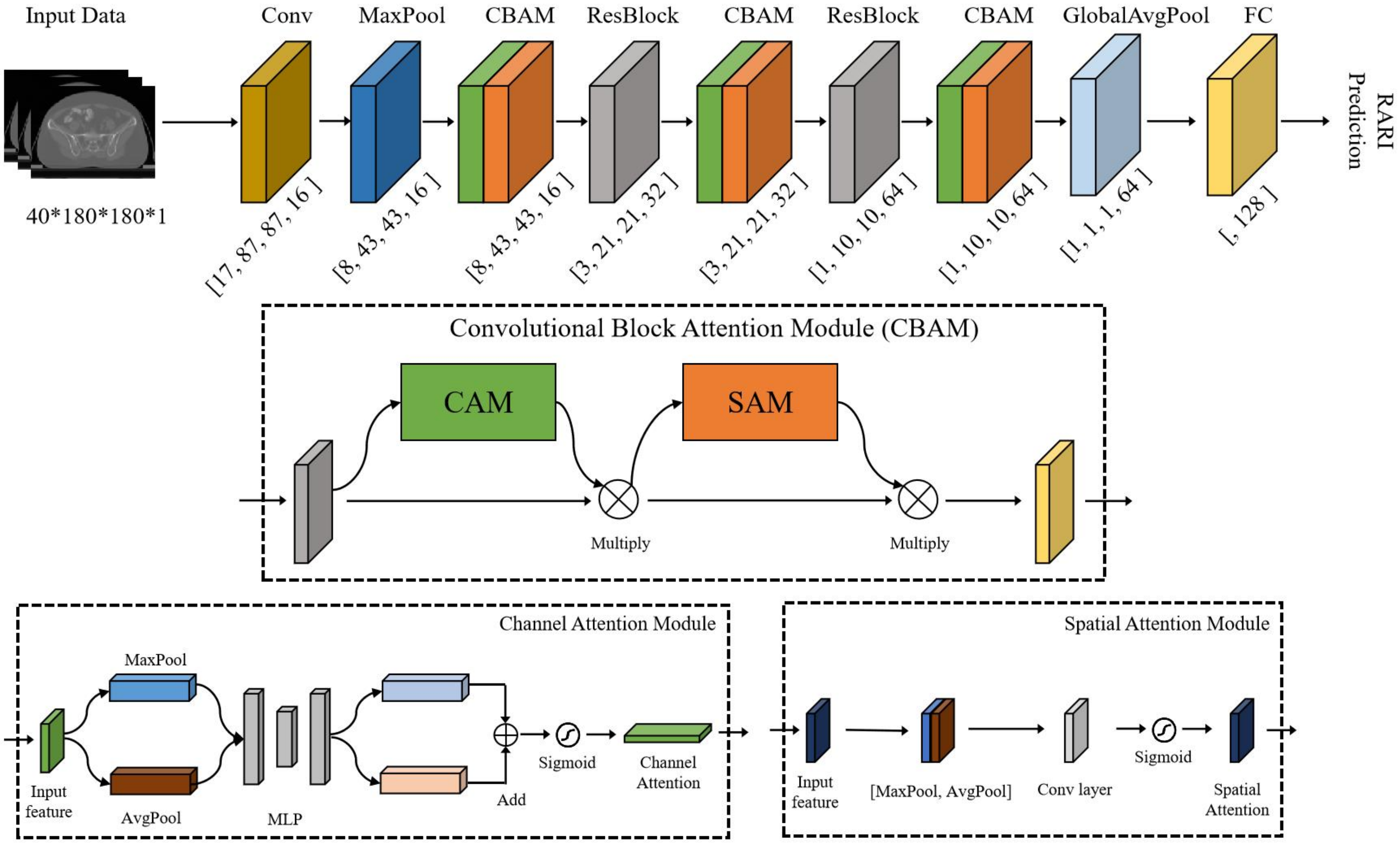

F. The details of the selected radiomics features and dosiomics features

Table F.1 The selected radiomics features based on CT.

ROI	Feature_Index	Features	P-Value
	16	original_firstorder_90Percentile	0.034
	570	wavelet-LLH_firstorder_Median	0.007
	717	wavelet-LHL_glszm_SizeZoneNonUniformityNormalized	0.021
	752	wavelet-LHH_firstorder_Median	0.024
	813	wavelet-LHH_glszm_ZonePercentage	0.014
Rectum	890	wavelet-HLL_glszm_GrayLevelNonUniformity	0.005
	999	wavelet-HLH_gldm_DependenceNonUniformityNormalized	0.007
	1148	wavelet-HHH_glrlm_GrayLevelNonUniformityNormalized	0.015
	1177	wavelet-HHH_glszm_ZonePercentage	0.001
	1199	wavelet-LLL_firstorder_90Percentile	0.037
	1222	wavelet-LLL_glcm_Correlation	0.035

Figure F.1 Radiomics feature selection using the LASSO regression.

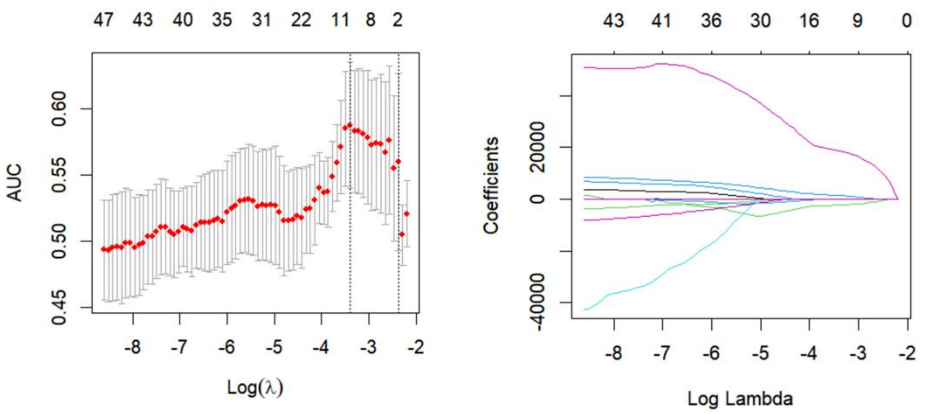

(a) (b)

(a) The percent deviance of variable quantity explained and (b) Tuning parameter (λ) selection by the LASSO logistic model in the rectum radiomics features.

Table F.2 The selected dosomics features based on Dose map.

ROI	Feature_Index	Features	P-Value
	309	log-sigma-3-0-mm-3D_glcm_ClusterShade	0.043
	358	log-sigma-3-0-mm-3D_glszm_ZonePercentage	0.039
	715	wavelet-LHL_glszm_LowGrayLevelZoneEmphasis	0.041
	1110	wavelet-HHH_firstorder_Entropy	0.029
	1123	wavelet-HHH_firstorder_Uniformity	0.029
Rectum	1148	wavelet-HHH_glrlm_GrayLevelNonUniformityNormalized	0.002
	1149	wavelet-HHH_glrlm_GrayLevelVariance	0.002
	1152	wavelet-HHH_glrlm_LongRunHighGrayLevelEmphasis	0.045
	1174	wavelet-HHH_glszm_SmallAreaHighGrayLevelEmphasis	0.025
	1184	wavelet-HHH_gldm_GrayLevelVariance	0.029
	1219	wavelet-LLL_glcm_ClusterShade	0.007

Figure F.2 Dosiomics feature selection using the LASSO regression.

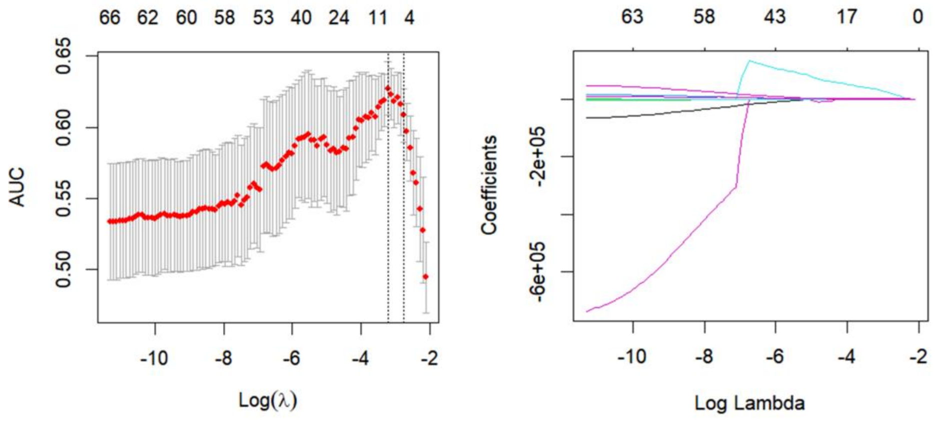

(a) (b)

(a) The percent deviance of variable quantity explained and (b) Tuning parameter (λ) selection by the LASSO logistic model in the rectum dosomics features.

G. The visualization of feature importance

Figure G.1 Visualization of radiomics features based on XGBoost model.

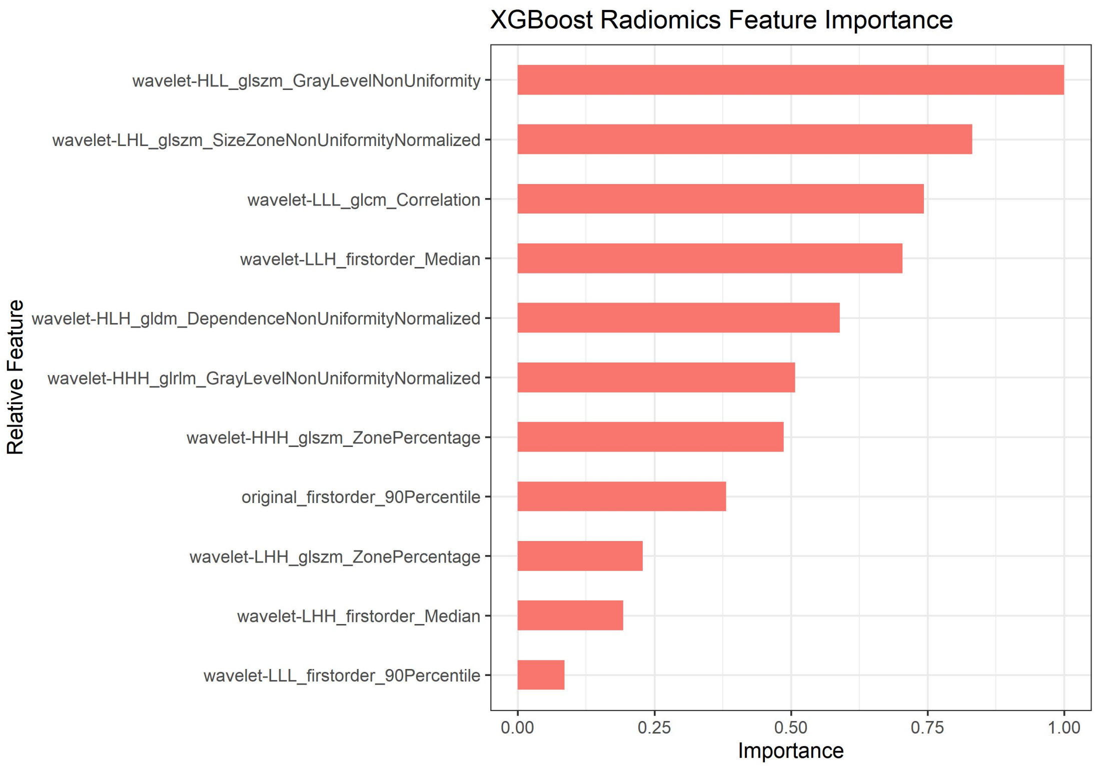

Figure G.2 Visualization of dosiomic features based on XGBoost model.

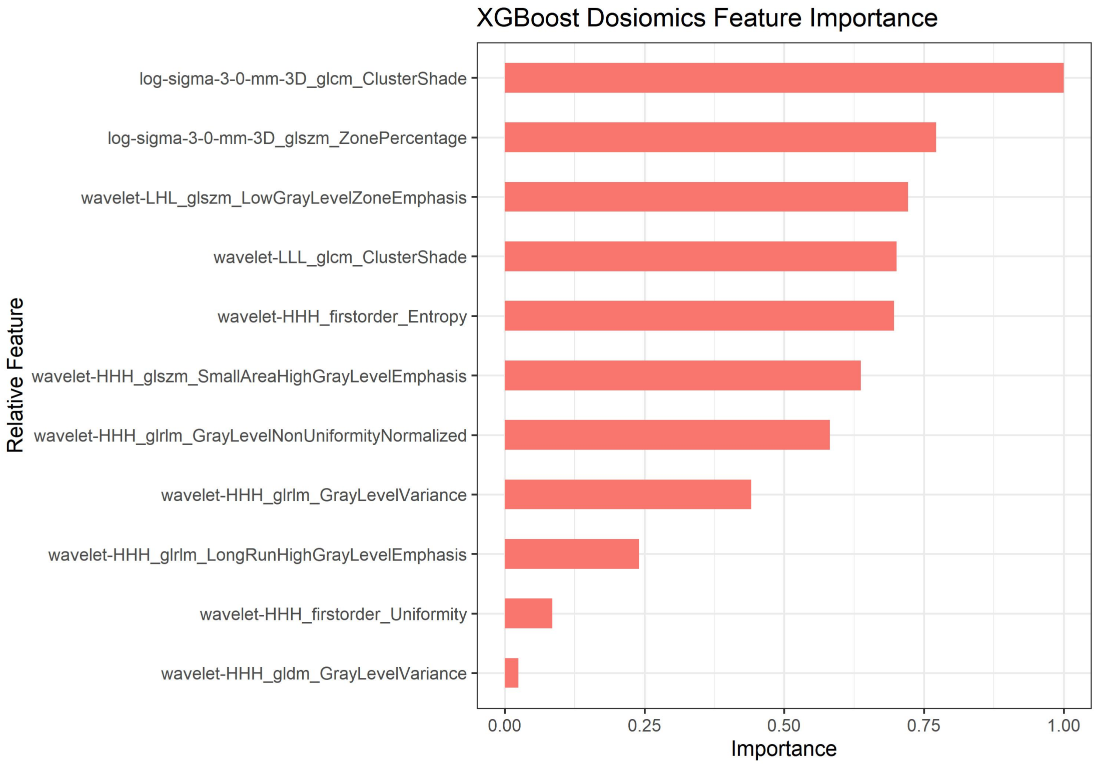

Figure G.3 Visualization of the patient examples. Feature heatmaps of representative patient generated from the Resnet_with_CBAM model based on the guided Grad-CAM.

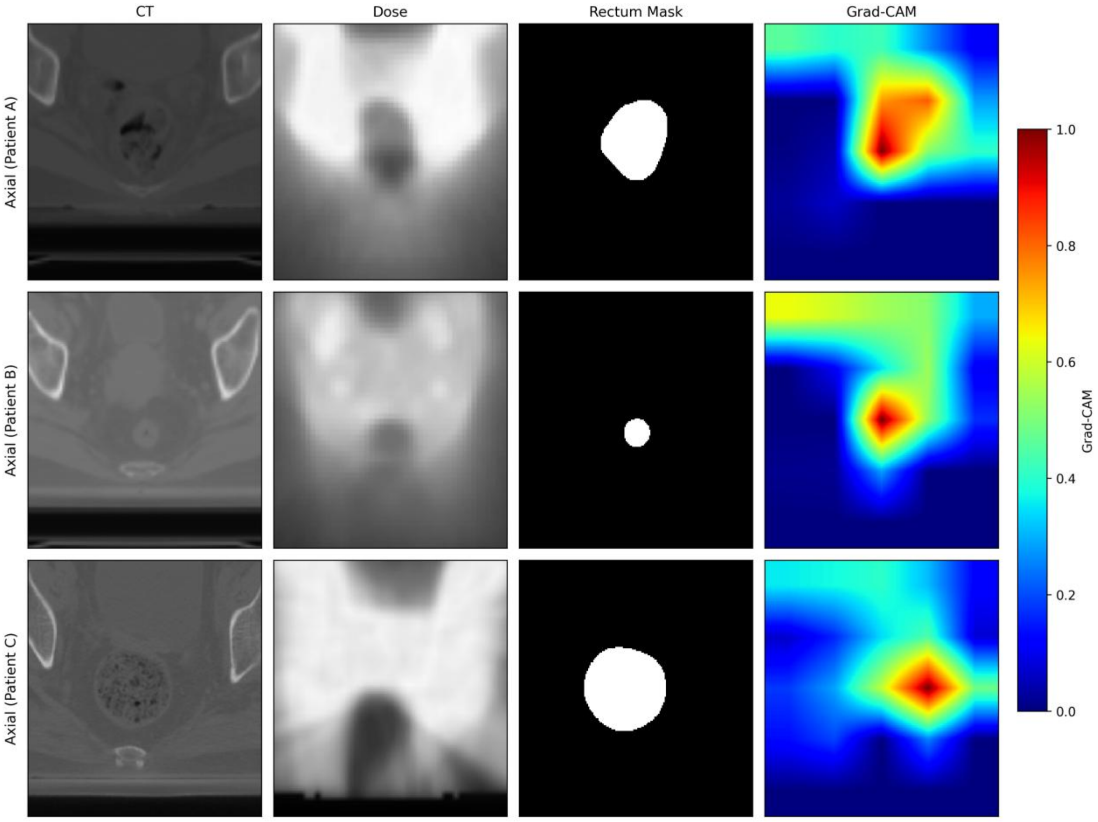

H. The performance of 11 deep learning models

Table H.1 The performance of 11 deep learning models based on CT and radiation dose distribution images

Input	Net Model	Training Cohort				Internal Validation Cohort				External Validation Cohort			
		AUC (95% CI)	ACC	Sen	Spe	AUC (95% CI)	ACC	Sen	Spe	AUC (95% CI)	ACC	Sen	Spe
DLR feature	Swin-Transformer	0.674 (0.582-0.758)	0.550	0.902	0.385	0.640 (0.455-0.808)	0.675	0.643	0.692	0.632 (0.459-0.813)	0.700	0.533	0.800
	SEnet	0.701 (0.622-0.780)	0.625	0.863	0.514	0.665 (0.490-0.835)	0.700	0.786	0.654	0.664 (0.457-0.853)	0.750	0.400	0.960
	Mobile-v2	0.755 (0.666-0.835)	0.787	0.608	0.872	0.690 (0.516-0.854)	0.600	0.929	0.423	0.693 (0.511-0.851)	0.725	0.667	0.760
	Mobile-v3	0.754 (0.667-0.837)	0.706	0.686	0.716	0.670 (0.497-0.845)	0.650	0.857	0.538	0.627 (0.429-0.803)	0.675	0.600	0.720
	Densenet-121	0.671 (0.584-0.758)	0.556	0.863	0.413	0.662 (0.476-0.827)	0.700	0.643	0.731	0.667 (0.489-0.849)	0.675	0.867	0.560
	Densenet-169	0.736 (0.654-0.809)	0.688	0.902	0.587	0.621 (0.437-0.797)	0.550	0.857	0.385	0.616 (0.416-0.795)	0.700	0.333	0.920
	Densenet-201	0.788 (0.710-0.855)	0.731	0.765	0.716	0.750 (0.580-0.896)	0.750	0.857	0.692	0.675 (0.484-0.849)	0.625	0.867	0.480
	Resnet-34	0.752 (0.677-0.826)	0.637	0.863	0.532	0.703 (0.516-0.865)	0.625	0.786	0.538	0.680 (0.508-0.848)	0.650	0.800	0.560
	Resnet-50	0.707 (0.623-0.789)	0.656	0.784	0.596	0.651 (0.446-0.840)	0.750	0.500	0.885	0.691 (0.506-0.857)	0.675	0.867	0.560
	Resnet-101	0.748 (0.676-0.829)	0.706	0.745	0.688	0.709 (0.547-0.869)	0.700	0.857	0.615	0.659 (0.478-0.838)	0.725	0.600	0.800
	Resnet_with_CBAM	0.827 (0.766-0.881)	0.744	1.000	0.624	0.739 (0.574-0.875)	0.675	0.929	0.538	0.728 (0.518-0.890)	0.750	0.600	0.840
DLD feature	Swin-Transformer	0.711 (0.626-0.783)	0.669	0.706	0.651	0.646 (0.466-0.815)	0.650	0.857	0.538	0.568 (0.393-0.744)	0.575	1.000	0.320
	SEnet	0.764 (0.684-0.836)	0.725	0.647	0.761	0.681 (0.486-0.832)	0.675	0.786	0.615	0.619 (0.442-0.783)	0.600	0.933	0.400
	Mobile-v2	0.760 (0.680-0.834)	0.706	0.804	0.661	0.646 (0.476-0.820)	0.625	1.000	0.423	0.680 (0.499-0.865)	0.725	0.600	0.800
	Mobile-v3	0.665 (0.575-0.753)	0.544	0.824	0.413	0.591 (0.403-0.784)	0.675	0.500	0.769	0.592 (0.396-0.790)	0.600	0.667	0.560
	Densenet-121	0.735 (0.644-0.823)	0.762	0.490	0.890	0.648 (0.458-0.815)	0.600	0.786	0.500	0.653 (0.448-0.857)	0.750	0.467	0.920
	Densenet-169	0.717 (0.635-0.795)	0.637	0.824	0.550	0.692 (0.509-0.840)	0.650	0.786	0.577	0.685 (0.483-0.867)	0.725	0.733	0.720
	Densenet-201	0.716 (0.628-0.796)	0.675	0.706	0.661	0.673 (0.487-0.840)	0.700	0.643	0.731	0.621 (0.429-0.805)	0.650	0.667	0.640
	Resnet-34	0.743 (0.661-0.816)	0.619	0.922	0.477	0.703 (0.536-0.850)	0.650	1.000	0.462	0.712 (0.519-0.871)	0.675	0.800	0.600

	Resnet-50	0.706 (0.614-0.788)	0.669	0.706	0.651	0.610 (0.385-0.803)	0.725	0.357	0.923	0.611 (0.423-0.789)	0.675	0.533	0.760
	Resnet-101	0.736 (0.653-0.813)	0.681	0.784	0.633	0.646 (0.458-0.815)	0.625	0.786	0.538	0.688 (0.516-0.854)	0.650	0.800	0.560
	Resnet_with_CBAM	0.774 (0.697-0.844)	0.713	0.961	0.596	0.728 (0.577-0.875)	0.750	0.857	0.692	0.685 (0.487-0.875)	0.725	0.600	0.800
DLR+DLD feature	Swin-Transformer	0.763 (0.690-0.829)	0.700	0.804	0.651	0.681 (0.519-0.844)	0.675	0.786	0.615	0.645 (0.452-0.821)	0.775	0.400	1.000
	SEnet	0.770 (0.693-0.843)	0.706	0.745	0.688	0.701 (0.520-0.859)	0.725	0.571	0.808	0.688 (0.500-0.846)	0.750	0.400	0.960
	Mobile-v2	0.820 (0.738-0.889)	0.838	0.882	0.817	0.717 (0.538-0.864)	0.650	0.857	0.538	0.688 (0.510-0.849)	0.725	0.533	0.840
	Mobile-v3	0.861 (0.798-0.914)	0.756	0.941	0.670	0.742 (0.556-0.900)	0.725	0.714	0.731	0.712 (0.544-0.872)	0.750	0.600	0.840
	Densenet-121	0.792 (0.728-0.859)	0.713	0.843	0.651	0.734 (0.573-0.883)	0.750	0.643	0.808	0.691 (0.480-0.880)	0.750	0.600	0.840
	Densenet-169	0.768 (0.690-0.838)	0.700	0.804	0.651	0.739 (0.558-0.890)	0.775	0.714	0.808	0.709 (0.504-0.896)	0.800	0.733	0.840
	Densenet-201	0.844 (0.773-0.905)	0.819	0.725	0.862	0.777 (0.624-0.918)	0.800	0.786	0.808	0.749 (0.578-0.887)	0.750	0.667	0.800
	Resnet-34	0.835 (0.768-0.897)	0.781	0.784	0.780	0.736 (0.568-0.875)	0.725	0.786	0.692	0.715 (0.538-0.878)	0.750	0.600	0.840
	Resnet-50	0.823 (0.752-0.877)	0.725	0.941	0.624	0.706 (0.522-0.856)	0.600	0.929	0.423	0.693 (0.494-0.860)	0.775	0.400	1.000
	Resnet-101	0.845 (0.784-0.902)	0.756	0.922	0.679	0.717 (0.539-0.857)	0.675	0.857	0.577	0.661 (0.486-0.828)	0.675	0.600	0.720
	Resnet_with_CBAM	0.862 (0.803-0.918)	0.787	0.941	0.716	0.786 (0.615-0.926)	0.750	0.857	0.692	0.773 (0.583-0.925)	0.800	0.867	0.760

Abbreviations: DLR feature: radiomics based on deep learning feature; DLD feature: dosiomics based on deep learning feature;

I. Univariate and multivariate analysis of clinical factors in predicting RARI

Table I.1 Univariate and multivariate analysis of clinical factors in predicting RARI

Characteristics	Univariate analysis			Multivariate analysis		
	P value	OR	95% CI	*P* value	OR	95% CI
Clinical factors						
Age	0.000	0.987	0.981-0.993	0.259	0.976	0.936-1.018
Diabetes	0.000	0.453	0.322-0.637	0.516	0.542	0.085-3.445
Hypertension	0.000	0.402	0.277-0.583	0.085	0.389	0.133-1.138
Surgery history	0.017	0.485	0.267-0.881	0.440	1.708	0.439-6.648
Treatment target	0.593	1.234	0.571-2.670	None	None	None
Targeted therapy	0.000	0.480	0.342-0.675	0.209	4.219	0.448-39.766
Immunotherapy	0.000	0.443	0.315-0.625	0.093	0.192	0.028-1.321
Pathological type	0.321	0.748	0.422-1.327	None	None	None
Chemotherapy regimen	0.000	0.696	0.576-0.841	0.582	1.134	0.724-1.776
Chemoradiotherapy regimen	0.021	0.732	0.562-0.953	0.257	1.294	0.829-2.020
Dose per fraction for EBRT(Gy)	0.000	0.996	0.994-0.998	0.293	0.960	0.889-1.036
Dose fractions for EBRT(Fx)	0.000	0.971	0.958-0.983	0.438	1.408	0.592-3.349
EBRT Total dose (Gy)	0.000	1.000	1.000-1.000	0.365	0.999	0.995-1.003
Rt technique	0.000	0.333	0.182-0.610	0.625	0.641	0.108-3.823
SIB	0.000	0.488	0.336-0.709	0.965	0.977	0.342-2.793
PTV (cc)	0.000	0.999	0.999-1.000	0.381	1.000	0.999-1.001
**BT applicator**	0.000	0.531	0.374-0.753	**0.033**	7.233	1.173-44.608
Source length	0.000	0.851	0.790-0.917	0.146	1.460	0.877-2.432
Dose per fraction for BT(Gy)	0.000	0.822	0.752-0.898	0.800	1.073	0.624-1.844
Dose fractions for BT(Fx)	0.000	0.879	0.830-0.930	0.223	14.144	0.199-1005.261
BT total dose (Gy)	0.000	0.968	0.953-0.982	0.215	0.632	0.306-1.306
DVH						
Rectum_Dmax (Gy)	0.000	1.000	1.000-1.000	0.208	0.997	0.992-1.002

Rectum_Dmean (Gy)	0.000	1.000	1.000-1.000	0.185	1.001	0.999-1.004
Rectum_V30 (%)	0.000	0.984	0.977-0.991	0.443	1.043	0.937-1.160
Rectum_V40 (%)	0.000	0.968	0.954-0.982	0.403	0.945	0.829-1.078
Rectum_D2cc (Gy)	0.000	1.000	1.000-1.000	0.666	1.001	0.996-1.007
Rectum_V_D2cc (%)	0.001	0.835	0.753-0.926	0.082	1.407	0.958-2.067

Abbreviations: RARI: radiation-induced acute rectal injury; EBRT: External beam radiotherapy; BT: Brachytherapy;
